# The State of Knowledge of the Primary and Secondary Metabolites of the *Iris* Genus

**DOI:** 10.1002/cbdv.202502203

**Published:** 2026-01-08

**Authors:** Olha Mykhailenko, Liudas Ivanauskas, Victoriya Georgiyants, Zigmantas Gudžinskas

**Affiliations:** ^1^ Pharmaceutical Chemistry Department National University of Pharmacy Kharkiv Ukraine; ^2^ Pharmacognosy and Phytotherapy Group UCL School of Pharmacy London UK; ^3^ Department of Pharmaceutical Biology Kiel University Kiel Germany; ^4^ Department of Analytical and Toxicological Chemistry Lithuanian University of Health Sciences Kaunas Lithuania; ^5^ Laboratory of Flora and Geobotany State Scientific Research Institute Nature Research Centre Vilnius Lithuania

**Keywords:** biogeography, chemotaxonomy, ecology, Iridaceae, pharmaceuticals

## Abstract

The genus *Iris* L. is one of the largest in the Iridaceae Juss. family, comprising more than 320 species. These plants are widespread across the Northern Hemisphere, particularly in temperate and subtropical climate zones. The greatest species diversity occurs in the Mediterranean region, as well as in Southwest and Central Asia. Throughout history, *Iris* species have had horticultural, cultural, and medicinal value. The rhizomes of several *Iris* species have long been used for treating various diseases and as a source of essential oils for the cosmetic industry. Phytochemical studies of plants have revealed a rich diversity of primary and secondary metabolites, including phenolic compounds, stilbenes, triterpenoids, quinones, amino acids, and organic acids. These specialised metabolites exhibit a wide range of biological activities, such as antitumor, antimicrobial, estrogenic, antioxidant, and other effects. Modern chromatographic and spectroscopic techniques have enabled precise structural characterisation and quantification of these compounds, thereby providing chemotaxonomic and pharmacological research. This review highlights the chemical diversity of key compounds in *Iris* species and examines the ecological and chemotaxonomic relationships underlying their distribution. This article aims to consolidate current phytochemical knowledge of the genus *Iris* and identify gaps in chemical analysis and ecological adaptation.

## Graphical abstract

The genus Iris L. (Iridaceae) comprises over 320 species, primarily found in the northern hemisphere. Iris species are important in horticulture, folklore and medicine, and contain a variety of primary and secondary metabolites with significant biological activity. This review summarises the latest phytochemical research on Iris species, considering their chemical diversity, chemotaxonomic significance, ecological patterns, and the current gaps in chemical and ecological research.

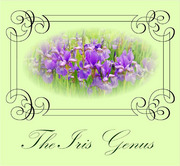

## 1 Introduction

The study of the diversity and resources of medicinal plants is currently a key topic in biodiversity research and conservation. Among them, plants of the genus *Iris* L. are an important component of plant diversity and value worldwide for their medicinal, ornamental, edible and technical applications.

The genus *Iris* is one of the largest in the Iridaceae family, which comprises about 1800 species distributed among 80 genera. Almost all *Iris* species are rhizomatous perennial herbs, often ephemeral, ranging in height from 5 to 200 cm. They display a wide variety of flower colours and possess significant ability for vegetative reproduction due to intensive rhizome branching.

*Iris* species are widespread throughout the Northern Hemisphere, particularly in temperate and subtropical regions. The largest species diversity occurs in the Mediterranean region, Southwest Asia and Central Asia. These plants usually grow in open, sunny habitats, although a few species are adapted to shaded or marshy areas. Many species exhibit high ecological plasticity and are classified as mesophytes, showing strong potential for successful cultivation and introduction to new environments.

Historically, *Iris* species have been cultivated as ornamental plants thanks to their striking floral morphology and bright colours. Additionally, essential oils extracted from the flowers have been used in perfumery, whereas the rhizomes have long been employed in traditional medicine [1]. The scientific name *Iris* was assigned by the Greek physician Hippocrates around the 4th century bce. The medicinal properties of *Iris* rhizomes were later documented by Dioscorides, Theophrastus, Aristotle and other classical physicians.

Ethnobotanical records highlight the use of *Iris* rhizomes as diaphoretic, expectorant and laxative agents, and in the treatment of liver and gallbladder disorders, cardiovascular diseases, venereal infections, ringworm, fever and oedema, as well as for blood purification [2]. The use of the underground parts of several *Iris* species was well established in traditional European medicine for centuries. The peeled and dried rhizomes of *Iris × germanica*, *Iris forentina* and *Iris pallida*, known collectively as *Rhizoma iridis*, were valued for their emetic, cathartic, stimulant, expectorant and errhine properties and were also used in tooth powders.

The rhizomes of *Iris* species are a rich source of secondary metabolites belonging to the classes of flavonoids, isoflavonoids, xanthones, stilbenes, simple phenolic compounds, triterpenoids, quinones and irons. These phytochemicals contribute to the diverse and beneficial properties of *Iris* extracts. The pharmacological activity of pure compounds and plant extracts from *Iris* species has been extensively described by Khatib [1], Singab [2] and Wang [3]. These plant extracts possess anti‐inflammatory, antioxidant, phytoestrogenic, antidiabetic, hepatoprotective, hypolipidemic, immunomodulating, antimutagenic, antimicrobial, antitumour, cytotoxic, antidepressant, anticholinesterase and molluscicidal effects. Recent studies have also highlighted the potential of *Iris* compounds and crude extracts against bacterial and viral infections, such as influenza H1N1 and enterovirus D68 [4]; amoebicidal activity against *Acanthamoeba* species [5], antiprotozoal potential [6] and cardioprotective activity [7]. Some *Iris* species have also been traditionally used as a natural dye for fabrics [8] and in perfumery [9].

Primary and secondary metabolites in *Iris* species include hundreds of compounds classified according to their biosynthetic origin. This review summarises the current state of research on the genus *Iris*, with focus on its phytochemical diversity and ecological significance. It also identifies promising future research directions, particularly in the discovery of natural products, conservation strategies and the sustainable use of *Iris* resources.

## 2 Materials and Methods

This review summarises data on the chemical constituents and biodiversity of species in the genus *Iris*. To compile all available information on the genus *Iris* in the public domain, keyword research was performed using the following search terms: *Iris species*, *phytochemistry*, *secondary metabolites*, *primary metabolites* and *environmental effect*. The databases included NCBI‐PubMed, Web of Knowledge, Google Scholar, ScienceDirect, Wiley Online Library, DOAJ and SpringerLink.

The taxonomy of *Iris* species was verified, and plant names were provided according to World Flora Online [10]. In cases of a change in taxonomic position, the species names used in the cited references are provided in synonymy (Table 1). We believe that a comprehensive analysis of the recent phytochemical profiling studies and the global distribution of species will enable natural product researchers to fully recognise the genus's potential.

**TABLE 1:** A list of the analysed *Iris* taxa, along with their main synonyms and native range.

Accepted name	Synonyms	Native range
*Iris adriatica* Trinajstic ex Mitic		Croatia
*Iris alberti* Regel		Central Asia
*Iris aphylla* L.	*Iris biflora* L., *Iris hungarica* Waldst. & Kit.	Central Europe, East Europe
*Iris auranitica* Dinsm.		Syria
*Iris barnumiae* Foster & Baker		Southwest Asia
*Iris bostrensis* Mouterde		Syria, Jordan
*Iris bracteata* S.Watson		North America
*Iris brevicaulis* Raf.		North America
*Iris bulleyana* Dykes		East Asia
*Iris bungei* Maxim.		East Asia
*Iris cathayensis* Migo		East Asia
*Iris chrysographes* Dykes	*Iris dykesii* Stapf	East Asia
*Iris chrysophylla* Howell		North America
*Iris clarkei* Baker ex Hook.f.		East Asia
*Iris confusa* Sealy		China
*Iris crocea* Jacquem. ex R.C.Foster	*Iris aurea* Lindl.	Himalaya
*Iris decora* Wall.	*Iris nepalensis* D.Don.	South Asia
*Iris delavayi* Micheli		East Asia
*Iris dichotoma* Pall.		East Asia
*Iris domestica* (L.) Goldblatt & Mabb.	*Belamcanda chinensis* (L.) Redouté; *Ixia chinensis* L.	East Asia, South Asia
*Iris douglasiana* Herb.		North America
*Iris ensata* Thunb.	*Iris kaempferi* Siebold ex Lem.	Asia
*Iris florentina* L.	*Iris albicans* Lange	Arabian Peninsula
*Iris foetidissima* L.		West Europe, North Africa
*Iris forrestii* Dykes		East Asia
*Iris fulva* Ker Gawl.		North America
*Iris × germanica* L.	*Iris × nepalensis* Wall. ex Lindl.; *Iris × sambucina* L.; *Iris × trojana* A.Kern. ex Stapf	Balkan Peninsula
*Iris gracilipes* A.Gray		Japan
*Iris graminea* L.		West Europe, Central Europe
*Iris halophila* Pall.	*Iris pallida* Salisb.	East Europe, West Asia
*Iris halophila* var. *sogdiana* (Bunge) Skeels	*Iris sogdiana* Bunge	Central Asia
*Iris haussknechtii* Bornm. ex Baker	*Iris kerneriana* Asch. & Sint. ex Dykes	Turkey
*Iris histrioides* (G.F.Wilson) S.Arn.		Turkey
*Iris × hollandica* H.R.Wehrh.		Hybridogenous species
*Iris hoogiana* Dykes		Central Asia
*Iris hookeriana* Foster		South Asia
*Iris humilis* Georgi	*Iris flavissima* Pall.	Asia
*Iris imbricata* Lindl.		Caucasus, Iran
*Iris japonica* Thunb.		East Asia
*Iris kashmiriana* Baker		Himalaya
*Iris kemaonensis* Wall. ex D.Don	*Iris kumaonensis* Auct.; *Iris tigrina* Jacquem. ex Baker	East Asia
*Iris lactea* Pall.		Asia
*Iris laevigata* Fisch.	*Iris phragmitetorum* Hand.‐Mazz.	East Asia
*Iris leptophylla* Lingelsh. ex H.Limpr.	*Iris sichuanensis* Y.T.Zhao	East Asia
*Iris loczyi* Kanitz	*Iris thianschanica* (Maxim.) Vved. ex Woronow & Popov	Central Asia, South Asia
*Iris lutescens* Lam.	*Iris longiflora* Vest; *Iris sordida* Willd.; *Iris subbiflora* Brot.	West Europe
*Iris marsica* I.Ricci & Colas.		Italy
*Iris milesii* Baker ex Foster		Central Asia, East Asia
*Iris missouriensis* Nutt.	*Iris montana* Nutt. ex Dykes	North America
*Iris nigricans* Dinsm.		Palestine
*Iris oxypetala* Bunge	*Iris lactea* subsp. *chinensis* (Fisch.) Kitag.	East Asia
*Iris pallida* Lam.		South Europe
*Iris paradoxa* Steven	*Iris medwedewii* Fomin	Southwest Asia
*Iris persica* L.		Southwest Asia
*Iris planifolia* (Mill.) T.Durand & Schinz	*Iris alata* Poir.	West Europe, North Africa
*Iris postii* Mouterde		Southwest Asia
*Iris potaninii* Maxim.	*Iris thorold* Barker ex Hemsl	Asia
*Iris pseudacorus* L.		Europe, West Asia
*Iris pseudopumila* Tineo		South Europe
*Iris pumila* L.	*Iris coerulea* Spach	East Europe, West Asia
*Iris reichenbachii* Heuff.		South Europe
*Iris reticulata* M.Bieb.	*Xiphion reticulatum* (M.Bieb.) Klatt	Southwest Asia
*Iris revoluta* Colas.		Italy
*Iris rossii* Baker		East Asia
*Iris sanguinea* Hornem.		East Asia
*Iris sanguinea* var. *sanguinea*		East Asia
*Iris sanguinea* var. *tobataensis* S.Akiyama & Iwashina		Japan
*Iris sari* Schott ex Baker		Turkey
*Iris scariosa* Willd. ex Link	*Iris elongata* Fisch. ex Baker	Asia
*Iris schachtii* Markgr.		Turkey
*Iris setina* Colas.		Italy
*Iris setosa* Pall. ex Link		North Europe, North Asia, North America
*Iris setosa* var. *hondoensis* Honda		Japan
*Iris setosa* var. *nasuensis* H.Hara		Japan
*Iris setosa* var. *setosa*		North Europe, North Asia, North America
*Iris sibirica* L.		Europe, West Asia
*Iris songarica* Schrenk ex Fisch. & C.A.Mey.		Central Asia, South Asia
*Iris spuria* L.	*Xiphion spurium* (L.) Alef.	Europe, Southwest Asia
*Iris spuria* subsp. *carthaliniae* (Fomin) B.Mathew	*Iris carthaliniae* Fomin	Caucasus
*Iris suaveolens* Boiss. & Reut.		South Europe, Turkey
*Iris susiana* L.	*Iris sofarana* Foster	Southwest Asia
*Iris taochia* Woronow ex Grossh.		Turkey
*Iris tectorum* Maxim.		China
*Iris tenuifolia* Pall.	*Iris acaulis* Pall.	East Europe, Asia
*Iris tingitana* Boiss. & Reut.	*Xiphion tingitanum* (Boiss. & Reut.) Baker	North Africa
*Iris unguicularis* Poir.		North Africa, Southwest Asia
*Iris variegata* L.		Europe
*Iris versicolor* L.		North America
*Iris virginica* L.		North America
*Iris wattii* Baker ex Hook.f.		Central Asia, Southeast Asia

## 3 Overview of *Iris* Taxonomy, Diversity and Distribution

**FIGURE 1:**
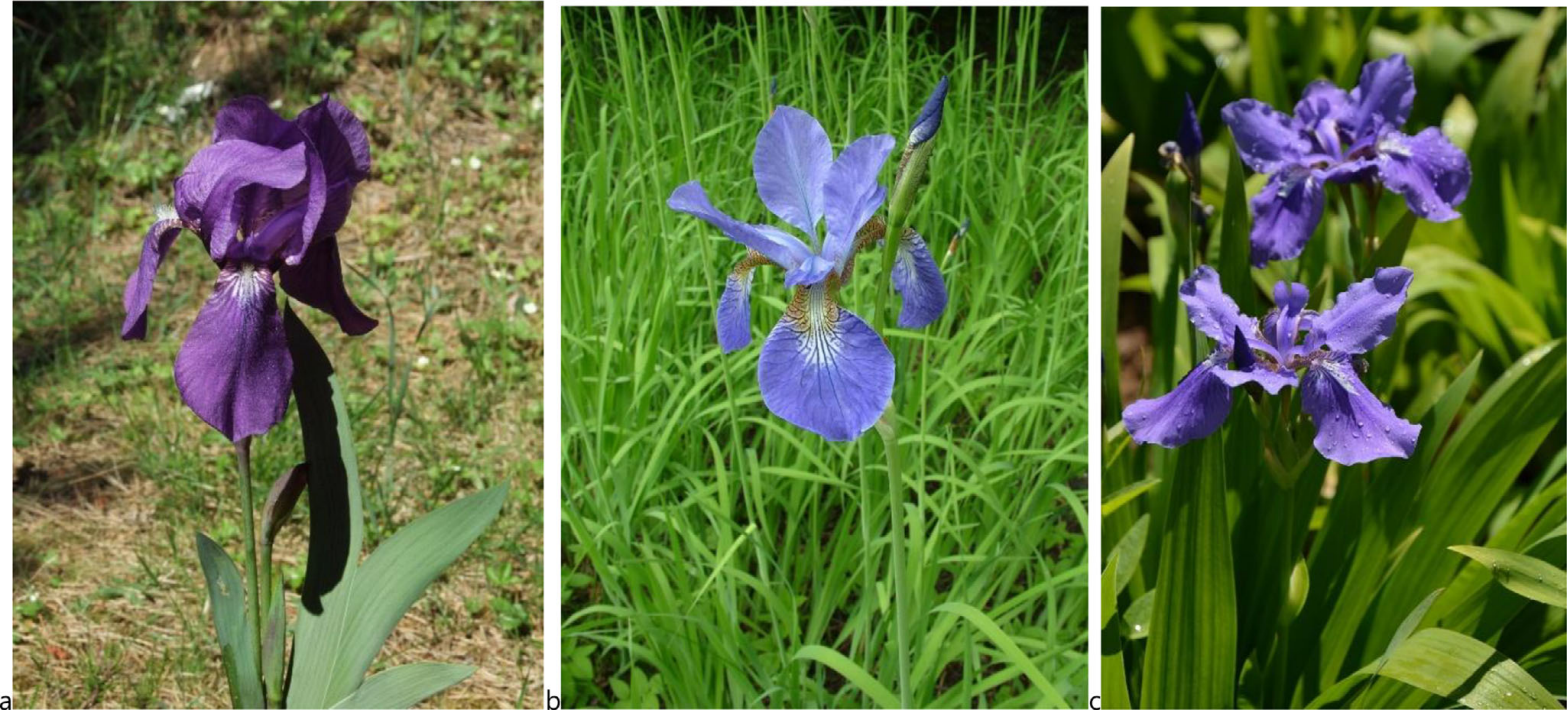
*Iris* species: (a) *Iris × germanica*, (b) *Iris sibirica* and (c) *Iris cristata*. *Source*: (a and b) Photos by Z. Gudžinskas. (c) Photo by Y. Buidin, Department of Ornamental Plants, M.M. Hryshko National Botanical Garden of the National Academy of Sciences of Ukraine, Kyiv, Ukraine.

The genus *Iris* (Fig. 1a‐c), which currently comprises around 320 species, is the largest in the Iridaceae Juss. family [10, 11, 12, 13]. The circumscription and taxonomy of the genus *Iris* have long been, and continue to be, a matter of discussion. Although some authors have adopted a broad concept of the genus [10, 13, 14, 15], others have divided it into as many as 25 narrowly defined genera [12, 16]. In this study, we follow the broad definition of the genus *Iris* [10] (Figure 1).

Mathew [14] developed the most comprehensive and widely accepted system for the broadly circumscribed genus *Iris*. In this classification, the genus was divided into six subgenera: *Iris* L., *Limniris* (Tausch) Spach, *Nepalensis* (Dykes) G. H. M. Lawrence, *Xiphium* (Mill.) Spach, *Scorpiris* Spach and *Hermodactyloides* Spach. Subsequent authors have revised and modified this system [17], but the molecular studies have not supported the division [15, 18, 19, 20, 21]. Further comprehensive molecular studies are necessary to resolve the remaining taxonomic issues within the genus [13]. Moreover, natural hybridisation significantly contributes to the taxonomic complexity of the highly diverse genus [22, 23].

The genus *Iris* is native to the Northern Hemisphere. Its most important centres of diversity are the Mediterranean region, Central Asia, East Asia and North America [10, 11, 13, 15]. The Mediterranean region harbours the highest number of species, with around 140 [24]. A total of 57 species have been found in the mountains of the Central Asian diversity centre [13], 58 in the East Asian diversity centre [25] and 34 in North America [26].

Most *Iris* species occur in arid regions, where they grow in semi‐deserts and on rocky mountain slopes. However, many species found in temperate areas grow in mesic habitats, such as grasslands, forests and forest edges, or even in wetlands [13, 23, 24, 26]. *Iris* species have been cultivated as ornamental plants for centuries, and tens of thousands of cultivars and hybrids have been developed to date [14, 24].

## 4 Characteristics of *Iris* Species

The genus *Iris* comprises perennial herbs with rhizomes, corms or bulbs. Different taxonomists sometimes classify plants with corms or bulbs as separate genera, whereas others include them in the genus *Iris* [12, 13, 14, 15, 16]. The height of the plants varies greatly. The rhizome is usually thick, horizontal and creeping, although in some species, it is thin and very short. The stem is typically single, but in some species, several stems may occur in clusters, either simple or branched. The leaves are flat and sword‐shaped, with a wax coating and arranged in two rows. The stem usually bears few leaves or may even be leafless.

The flowers are large and may occur singly or in small clusters on long peduncles. The inflorescence is enclosed by one to three elongated, pointed, sometimes inflated, leaf‐like bracts. The perianth of the flower consists of six parts arranged in two rows: The three outer segments (sepals) spread out or droop downwards, whereas the three inner segments (petals) stand upright. The three styles in *Iris* flowers are large and resemble petals. These distinctive floral features make it easy to recognise whether a plant belongs to the genus *Iris*.

The flowers of *Iris* species are pollinated by insects, most commonly bees and bumblebees attracted by the nectar. The outer segments of the perianth serve as landing platforms for pollinators. *Iris* species typically flower from May to July, and the buds of the following year's flowers are formed in the rhizome during summer. The fruit is a multi‐seeded syncarpous capsule that dehisces loculicidally along the midrib of each of the three carpels. The seeds are either flattened or nearly spherical, with a membranous or papery coat, and sometimes bear a distinct appendage.

## 5 Bioactive Compounds in Plants of the *Iris* Genus

Since the beginning of research on the *Iris* genus, several reviews have been published on the distribution of secondary metabolites in these plants [1, 27, 28]. One of the most comprehensive reviews of the phenolic compounds of the Iridaceae family was conducted in the 1980s by Williams et al. [29, 30]. Later, in 1997, the same authors focused predominantly on flavonoids, isoflavones and xanthones, recording 17 glycosylflavones in 14 *Iris* species, with the aim of tracing chemical evolution within the genus [31].

In 1998, Donnelly and Boland [32] summarised data on more than 260 new isoflavonoid compounds isolated from plants, including species of the genus *Iris*, between 1991 and 1996. A detailed analysis of the chemical composition of *Iris* species was also published by Iwashina in the same year [33], describing the structure, distribution and functions of 46 different isoflavonoid aglycones from 18 *Iris* species, predominantly from China.

In 2005, French scientists [34] published a review on the distribution of isoflavones in plants. Among monocotyledonous taxa, *Iris* species exhibited the highest diversity of isoflavonoids, with 52 compounds recorded.

Chinese scientists [3] later summarised data on flavonoids and isoflavonoids of *Iris* species published between 1999 and 2008. By 2010, 56 isoflavones had been identified, 20 of which were new compounds (5 isoflavones and 15 isoflavone glycosides).

Between 2012 and 2015, Polish and Czech researchers [35, 36] compiled information on the distribution of bioactive compounds and pharmacological activity of various *Iris* species. These plants were found to contain flavonoids, isoflavonoids, xanthone C‐glycosides, quinones and iridal‐type triterpenoids [37, 38]. In 2016, Singab et al. [2] published a review of plants in the Iridaceae family, focusing on isoflavonoids, flavonoids, triterpenoids, quinones, xanthones and simple phenolics in the *Iris* genus. The authors described 58 isoflavones.

The most recent and comprehensive review of the chemical composition of *Iris* species was published in 2020 by Iwashina and Mizuno [39]. Between 1961 and 2020, 85 isoflavonoids, 84 flavonoid aglycones and glycosides, 6 benzenecarboxylic acids, 7 phenylpropanoids, 6 anthocyanidins, 9 aceto‐ and benzophenones, 6 stilbenes and their derivatives and 15 xanthones were isolated and identified from 90 taxa of the genus *Iris*, including flowers, leaves and rhizomes. This review covered approximately 32% of *Iris* species distributed worldwide. Chalcones, dihydrochalcones, aurones, bioflavonoids and neoflavonoids have not been reported in the *Iris* genus.

One of the most recent reviews by Khatib [1] focuses primarily on the ethnobotanical and ethnoveterinary uses of *Iris* species, as well as evaluating their pharmacological potential. It also describes the chemical composition of *Iris* extracts, including phenolic acids, flavonoids, alkaloids, primary metabolites and essential oil components.

In addition to general reviews, several studies have been devoted to the chemical composition and pharmacological potential of individual *Iris* species, particularly *I*. *ensata* [40], *I*.* × germanica* [27], *I*. *kashmiriana* [41] and *I*. *domestica* [42, 43].

**FIGURE 2:**
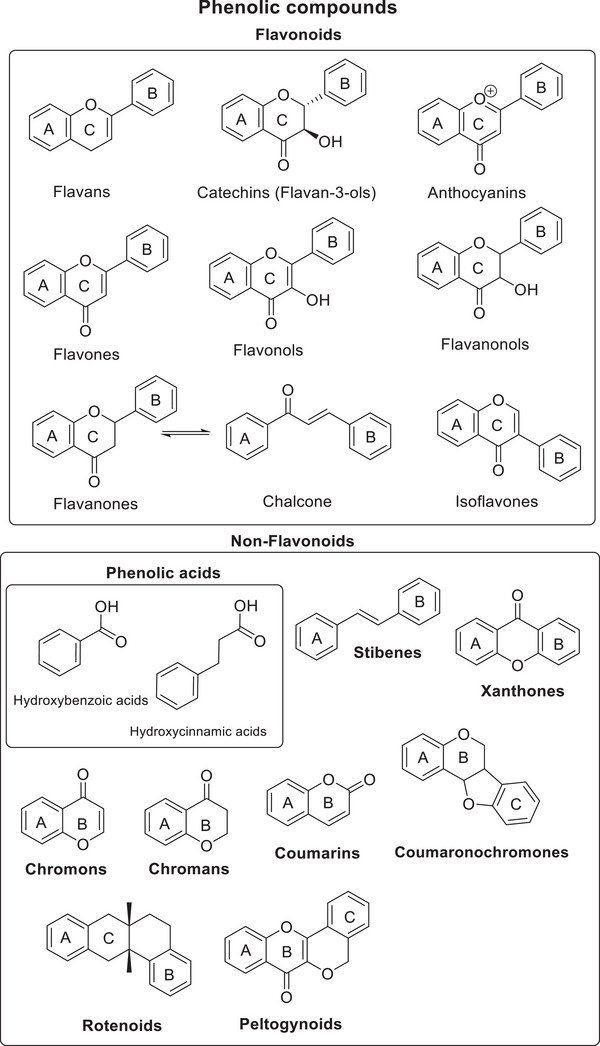
Classification of phenolic compounds.

This present review summarises and integrates the findings of these authors while providing an updated overview of compounds identified in *Iris* species in recent years. Special attention is given to phenolic compounds, which are classified into six major classes: isoflavonoids and isoflavone glycosides; flavonoids and flavone glycosides; peltogynoids; coumaronochromones; rotenoids; and xanthones and phenol carboxylic acids. This classification is illustrated in Figure 2. Additionally, the review considers other classes of bioactive compound found in *Iris* plants, including terpenoids, amino acids, carboxylic acids, alkaloids and essential oils.

Isoflavones and flavones in *Iris* species typically contain simple O‐substituents, such as hydroxy‐, methoxy‐ and methylenedioxy groups, as well as sugar components. The substitution pattern differs between rings A and B: phenolic compounds most often exhibit 5,7‐di‐ or 5,6,7‐tri‐*O*‐substitution in ring A, whereas ring B often has 2′‐ or 4′‐*O*‐substitution, as well as 2′,3′‐ or 3′,4′‐di‐*O*‐substitution, and occasionally 3′,4′,5′‐tri‐*O*‐substitution. It is noteworthy that the aglycones of their glycosides rarely display 2′‐*O*‐ or 2′,3′‐di‐*O*‐substitution patterns.

In addition to typical flavonoids, *Iris* species also contain structurally modified phenolic compounds formed through cyclisation of flavonoid derivatives. These include peltoquinoids, coumaronochromones and protenoids. Similar to conventional flavonoids, they possess simple O‐substituents. However, a characteristic feature of these compounds is the presence of an oxygen‐containing ring condensed with rings B and C, typically involving substitution at the C‐2 or C‐3 position.

### 5.1 Isoflavonoids and Isoflavone Glycosides

**FIGURE 3:**
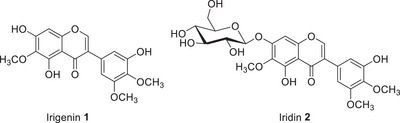
Structural formulas of irigenin **1** and iridin **2** as the most common compounds identified in *Iris* species.

Plants of the genus *Iris* contain a wide range of compounds belonging to different classes and exhibiting diverse pharmacological activities. The underground organs predominantly accumulate isoflavones, xanthones, essential oils and triterpenoids, whereas the aerial parts are rich in flavonoids, polyphenolic acids and xanthones. The first isoflavonoids isolated from the rhizomes of *Iris florentina* were the aglycone irigenin **1** and its 7‐glucoside, iridin **2** (irigenin 7‐*O*‐β‐d‐glucopyranoside). Irigenin, detected in 23 *Iris* species [39], and iridin, identified in 16 species, are among the most widely distributed compounds within the genus (Figure 3).

Isoflavones and their glycosides are most often found in the rhizomes of *Iris* species and, to a lesser extent, in the leaves and flowers. Analysis of the distribution of phenolic compounds in the *Iris* genus indicates that *I*.* × germanica* has been the most extensively studied, followed by *I*. *tectorum*, *I*. *florentina* and *I*. *dichotoma*.

**FIGURE 4:**
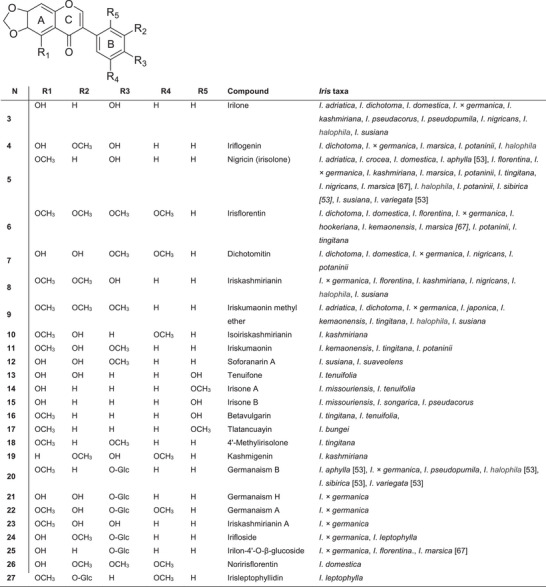
Structures of the most common isoflavone derivatives containing a methylenedioxy group identified in *Iris* species. The distribution of these compounds follows the classification by Iwashina and Mizuno [39], with additional data from recent studies [53, 67].

The most common isoflavones in this genus are tectorigenin and its glycosides, which have been identified in 20 species (Figure 4). Of these, six belong to subgenus *Iris*, four to subgenus *Crossiris*, six to subgenus *Limniris*, three to subgenus *Xyridion* and one to subgenus *Parandanthopsis*. Irilon has been reported in 12 species, and nigricin in 14 species. Tectorigenin and irigenin contain hydroxyl groups At Positions 5 and 7, as well as methoxy groups at Position 6, whereas nigricin and irilon feature a methylenedioxy group at the 6,7‐position of ring A.

Isoflavonoids containing a methylenedioxy group in ring A are relatively rare and are unevenly distributed in nature. The Iridaceae family is the largest source of isoflavonoids, with more than 50 distinct compounds described. Figure 4 shows the most common isoflavones containing a methylenedioxy group. The largest number of isoflavonoids with a 6,7‐methylenedioxy group (32 out of 94 isoflavonoids) has been isolated from *Iris* species, most of which belong to the subgenus *Iris*. Irilone **3** was found in eight species of subgenus *Iris*, three species of subgenus *Limniris* and one species of subgenus *Pardanthopsis*. The highest number of isoflavonoids with a 6,7‐methylenedioxy group and their glycosides were isolated from the rhizomes of *Iris potaninii* (nine compounds) and *I*.* × germanica* (14 compounds). Among the most notable are iriflogenin **4**, nigricin (or irisolone) **5**, irisflorentin **6**, dichotomitin **7**, nigricanin (or iriskashmirianin) **8** and iriskumaonin methyl ether **9**.

A distinctive compound of the genus *Iris* is irisolone **5** (also known as nigricin), which was first isolated from the rhizomes of *I*. *florentina* [44] and later from *Iris nigricans* [45]. In addition to nigricin, the isoflavonoids iriskashmirianin **8** and irilone **3**, belonging to the 5,6,7‐trihydroxy group of isoflavones, were also isolated from the rhizomes of *I*. *nigricans*. Nigricin was found in nine species from subg. *Iris*, one from subg. *Limniris* and one from subg. *Xyridion*.

An example of an isoflavone found in *Iris* plants with hydroxy or methoxy substituents at the 3′,4′‐ or 3′,4′,5′‐tri‐O‐positions of ring B is sophoronarin A **12**. This compound was first isolated from the methanol extract of the rhizomes of *Iris susiana* (subg. *Iris*) from Turkey, together with sophoronarin B [46]. Iristectorigenin B (5,7,4′‐trihydroxy‐6,4′‐dimethoxyisoflavone) was initially isolated from *I*. *florentina* rhizomes [44] and was subsequently identified in *Iris pseudacorus*, *Iris spuria* subsp. *carthaliniae*, *I*. *tectorum* and *I*.* × germanica*. Iristectorigenin A (5,7,3′‐trihydroxy‐6,4′‐dimethoxyisoflavone), which contains 3′,4′‐substituents in ring B, was found in eight *Iris* species.

Functional groups in the B ring are most commonly found at the C‐4′ position. However, some species of subgenus Limniris are characterised by isoflavonoids containing a hydroxyl or methoxyl group at the 2′‐position of the B ring. Species‐specific 6,7‐methylenedioxy isoflavonoids isolated from *Iris tenuifolia* include tenuifone **13** (5,2′,3′‐trihydroxy), tenuifodione, irisone B (5,2′‐dihydroxy‐) **15** and betavulgarin (6′‐hydroxy‐5‐methoxy‐) **16** [47]. Irilins A and B, along with 5,7‐dihydroxy‐6,2′‐dimethoxyisoflavone, were also identified in the rhizomes of *Iris songarica*.

A large number of unique isoflavonoids have been isolated from *Iris* species. Tlatancuyain (5,2′‐dimethoxy‐6,7‐methylenedioxyisoflavone) **17** was isolated from the rhizomes of *Iris bungei* [48]; irisolone (4′‐*O*‐[*O*‐β‐d‐glucopyranosyl‐(1→6)‐*O*‐β‐d‐glucopyranosyl‐(1→6)‐β‐d‐glucopyranoside) and iriskashmirianin (4′‐*O*‐[*O*‐β‐d‐glucopyranosyl‐(1→6)‐*O*‐β‐d‐glucopyranosyl‐(1→6)‐β‐d‐glucopyranoside) were isolated from the underground parts of *I*. *florentina* [49]. Additionally, 5,6‐dihydroxy‐7,8,3′,5′‐tetramethoxyisoflavone was identified in the rhizomes of *I*. *pseudacorus* [50] and has also been detected in *Iris halophila*, *Iris aphylla*, *Iris sibirica*, *Iris variegata* and *I*.* × germanica* [51, 52]. Other common occurring isoflavones include tectoridin, irigenin, iristectorigenin B, irisolidone, irisolidone‐7‐*O*‐β‐d‐glucopyranoside and nigricin [53].

New isoflavones containing a methylenedioxy group at the 6,7‐position of ring A include the aglycone iriskashmirianin A **23** and the glycoside germanaism H **21**, both first isolated from the rhizomes of *I*.* × germanica* [54]. In addition, eight known isoflavones were also isolated: irilone, iriskumaonin methyl ether, iriflogenin **4**, irifloside **24**, irilon‐4′‐*O*‐β‐glucopyranoside **25** and germanaisms A–B **20** and **22**. Isoflavonoids with an oxygen‐containing functional group at the 2′‐position are uncommon in species of subgenus *Iris*, with *I*.* × germanica* being a notable exception. Noririsflorentin (5‐hydroxy‐6,7‐methylenedioxy‐3′,4′,5′‐trimethoxyisoflavone) **26** has been isolated only once, from *I*. *domestica*. Two unusual isoflavone monosaccharides have also been identified: irisleptophyllidin **27** from the rhizomes of *Iris leptophylla* [55] and 7,4′‐dimethoxy‐8,3′,5′‐trihydroxy‐6‐*O*‐β‐d‐glucopyranosylisoflavone [56], isolated from the underground parts of *I*. *potaninii* and structurally identified as 3′‐*O*‐ and 6‐*O‐*β‐d‐glucopyranosides, respectively.

From the ethyl acetate fraction of the rhizomes of *I*. *spuria* subsp. *carthaliniae* [57], three isoflavonoid glycosides were isolated for the first time: tectorigenin 4′‐glucosyl(1→6)‐glucoside, iristectorigenin B 7‐glucosyl(1→6)‐glucoside and 4′‐methyltectorigenin 7‐glucoside. Tectorigenin glucosides have also been found in the rhizomes of *Iris confusa* and *I*. *dichotoma* [58], as well as in the rhizomes of *I*. *variegata*, *Iris milesii*, *I*. *tectorum*, *I*. *leptophylla*, *I*. *songarica*, *I*. *spuria*, *Iris crocea* and *Iris cathayensis* [30]. Iristectorins A and B have been found in *I*. *spuria*, *I*. *tectorum* and *I*. *dichotoma* [58, 59, 60].

New mono‐ and diglycosides of germanaisms A–E and G–H, which differ in the position of the aglycone, as well as the only triglycoside of germanaism F, were isolated from the rhizomes of *I*.* × germanica* using column chromatography on silica gel. Notably, germanaism G [61, 62] is 3′,4′‐di‐*O‐*β‐d‐glucopyranosylisoflavone and contains a 2‐methoxy‐4‐acetylphenyl glucose derivative at the C‐2 position of the 4′‐glucopyranoside. This rare phenyl glucose residue is exceedingly uncommon among isoflavone glycosides of the *Iris* genus. Germanaisms A, B and E also have been identified in *Iris pseudopumila* [63] as well as in *I*. *leptophylla*, *Iris marsica* and *I*. *florentina*, all belonging to subgenus *Iris*.

Other glycosides isolated from *Iris* species include iriskashmirianin 4′‐*O*‐β‐d‐glucoside, irilon 4′‐*O‐*β‐d‐glucoside, irisolidon 7‐*O‐*α‐d‐glucoside, irigenin 7‐*O*‐glucoside, irisolon‐4′‐*O*‐bioside and irilon‐bioside. The specific isoflavone muningin (6,4′‐dihydroxy‐5,7‐dimethoxyisoflavone) has only been identified in *I*.* × germanica* [64].

New isoflavonoid glucosides, including iridins B–C, tectoridin A and ampelopsinin A, along with one previously undescribed phenolic glucoside (diplostephioside B) and one novel phenolic compound (phenanthrenetriol A), were isolated from the rhizomes of *I*. *domestica* [65]. Isoflavanones, which are widespread in the Fabaceae and Vitaceae families, are rare in the genus *Iris*, with the only reported example being 2,3‐dihydroirigenin from *I. halophila* [66].

Although isoflavonoids are among the most studied secondary metabolites in the genus *Iris*, their structural diversity across subgenera remains incompletely characterised. Many of the identified compounds, especially in poorly studied ones, are promising for further analysis aimed at clarifying chemosystematic relationships within the genus.

### 5.2 Flavones and Glycosides of Flavones

**FIGURE 5:**
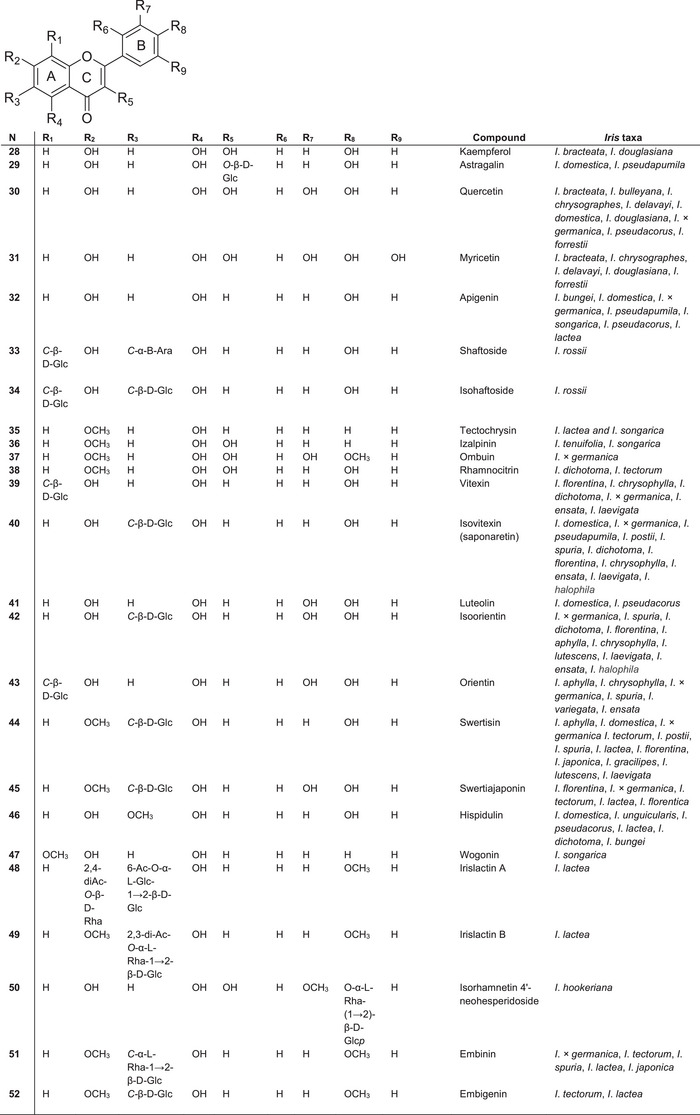
The most abundant flavones found in plants of the *Iris* genus.

As described by Iwashina [33] the main flavonoid components of *Iris* plants are C‐glycosylflavones (such as vitexin **40**, isovitexin **41**, isorientin **42,** orientin **43** and swertisin **44**) and *O*‐glycosylflavones, which occur mainly in the leaves and rhizomes of most species of *Iris*. Mono‐ and di‐C‐glycosides of apigenin, luteolin, quercetin and myricetin are also commonly present (Figure 5).

In contrast to 7‐ and 4′‐*O*‐glycosyl isoflavones, the sugar moieties in these compounds are typically attached at the C‐6 and C‐7 positions of the aglycone. Unlike isoflavones, flavones in *Iris* species predominantly occur as 6‐C‐glycosides. For example, isorhamnetin 4′‐neohesperidoside **50** and isovitexin **40** have been reported in species of subgenus *Limniris* (*Iris rossii*, *I*. *ensata*) and subgenus *Iris* (*I*. *pseudopumila*, *I*. *florentina*) [31, 68, 69]. Along with the flavon glycosides embinin **51** and embigenin **52**, two unusual acetylated C‐glycosylflavone derivatives—irislactin A **48** and irislactin B **49**—were isolated from the leaves of *Iris lactea* (subg. *Limniris*, sect. *Limniris*) [70]. These compounds differ in their substituents and the glucosylation pattern at the C‐6 and C‐7 positions of ring A.

Irislactin A **48** is an acetylated linear disaccharide, identified as 7‐*O*‐(β‐d‐2⁗,4⁗‐diacetylrhamnopyranosyl)‐6‐C‐[*O*‐(α‐l‐6‴‐acetylglucopyranosyl)‐1→2‐β‐d‐glucopyranoside] 5‐hydroxy‐4′‐methoxyflavone [71]. Irislactin B **49** is also an acylated linear disaccharide with a methoxyl group at C‐7 of ring A, that is, 6‐С‐[O‐(α‐l‐2‴,3‴‐diacetylrhamnopyranosyl)‐1→2‐β‐d‐glucopyranoside] of 5‐hydroxy‐7,4′‐dimethoxyflavone [72]. C‐glycosides of flavones (6‐С‐(O‐α‐l‐2‴‐acetylramnopyranosyl‐1→2‐β‐d‐glucopyranoside) 5‐hydroxy‐7,4′‐dimethoxyflavone and 6‐С‐(O‐α‐l‐3‴‐acetylramnopyranosyl‐1→2‐β‐d‐glucopyranoside) 5‐hydroxy‐7,4′‐dimethoxyflavone) were isolated from *I*. *tectorum* [73]. These compounds contain one fewer acetyl group than irislactin B. In *I*. *rossii*, the C‐glycosylflavone shaftoside **33** and isoshaftoside **34** were also identified [74].

The most unique flavonoid identified in the genus *Iris* is irisflavone D, which has 2′,6′‐di‐*O*‐substitution in the B‐ring, a feature that is rare among *Iris* flavonoids. This compound was first isolated from the rhizomes of *I*. *bungei* [75]. Irisflavone A was also isolated from the related species *I*. *songarica*. Flavones containing a methylenedioxy group at the C‐6 and C‐7 positions of ring A, such as kanzakiflavone‐1 and kanzakiflavone‐2, were isolated from the rhizomes of *Iris unguicularis* [76]. These two compounds differ in the position of hydroxy groups, occurring at 5,8‐OH and 5,4′‐OH, respectively.

Among the newly identified flavones, 5,2′‐dihydroxy‐3‐methoxy‐6,7‐methylenedioxyflavone, isolated from the rhizomes of *I*.* × germanica*, is notable for its 2′‐*O*‐substitution in ring B. This is another characteristic feature of *Iris*‐derived flavonoids. Between 1999 and 2008, only two new flavone aglycones were isolated: tectochrysin **35** from *I*. *lactea* and *I*. *songarica* [70] and wogonin **47** from *I*. *songarica* [77]. The novel flavonol izalpinin **36** was isolated from *I*. *tenuifolia* [47] and *I*. *songarica* [77].

The flavone ombuin **37** (4′,7‐dimethoxy‐3,3′,5‐trihydroxyflavone) was first isolated from the rhizomes of *I*.* × germanica*, marking its initial discovery within the Iridaceae family [64]. The flavonol rhamnocitrin **38** was isolated from the rhizomes of *I*. *dichotoma* and *I*. *tectorum* [78]. The latest investigation by Kai‐Dong Liu [59] described several new flavonoid glucosides, such as iridins B–C, tectoridin A and ampelopsinin A, along with one previously undescribed phenolic glucoside (diplostephioside B) and a new phenolic compound (phenanthrene triol A), isolated from the rhizomes of *I*. *domestica*.

The most recent analysis (2024) led to the isolation of five new flavonoids from the underground parts of *I*. *tenuifolia*, all bearing 2,3‐ and 3,4‐dihydroxy substituents on the phenyl ring [79].

Current knowledge of flavonoids in *Iris* species highlights the occurrence of both common and structurally unique flavones, especially in the form of C‐glycosides. However, there remains a significant gap in understanding the regulation of species‐specific flavone biosynthesis and their functional role in stress adaptation. These aspects could be clarified through targeted metabolomic and transcriptomic analysis. Due to the current lack of such data, these topics were not addressed in this review.

### 5.3 Flavanones and Dihydroflavonols

**FIGURE 6:**
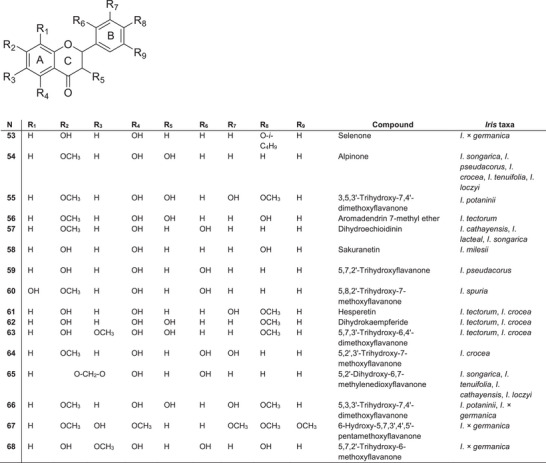
The most abundant flavanones and dihydroflavonols found in plants of the *Iris* genus.

Flavanones are a prominent class of secondary metabolites in the *Iris* genus, notable for their structural diversity and chemotaxonomic significance. The flavanones identified across different *Iris* species exhibit variations in hydroxylation and methoxylation patterns, particularly on the A‐ and B‐rings (Figure 6). Several novel or rare compounds have been identified, including selenone **53** from *I*.* × germanica* [80], alpinone **54** from *I*. *songarica* rhizomes [77], 3,5,3′‐trihydroxy‐7,4′‐dimethoxyflavanonol **55** from *I*. *potaninii* [56] and aromadendrin 7‐methyl ether **56** from *I*. *tectorum* rhizomes [81]. The dihydroflavanonol alpinone **54** has been found in four species of subgenus *Limniris* (*I*. *pseudacorus*, *I*. *crocea*, *I*. *tenuifolia* and *Iris loczyi*) [39] but is absent in species of subgenus *Iris*. Dihydroechioidinin **57** was first reported in *I*. *cathayensis* rhizomes [82] and later detected in *I*. *lactea* and *I*. *songarica* [3].

The flavanone sakuranetin **58** was uniquely isolated from *I*. *milesii* [83], whereas 5,7,2′‐trihydroxyflavanone **59** and 5,8,2′‐trihydroxy‐7‐methoxyflavanone **60** were identified in *I*. *pseudacorus* [84] and *I*. *spuria* [85], respectively. Hesperetin **61**, dihydrokaempferide **62** and 5,7,3′‐trihydroxy‐6,4′‐dimethoxyflavanone **63** were reported in *I*. *tectorum* [78] and *I*. *crocea* [85]. Furthermore, Bhat et al. [86] identified 5,2′,3′‐trihydroxy‐7‐methoxyflavanone **64** in the methanol extracts of *I*. *crocea* rhizomes.

The presence of 5,2′‐dihydroxy‐6,7‐methylenedioxyflavanone **65** has been confirmed in four species of section *Tenuifoliae*: *I*. *songarica* [77], *I*. *tenuifolia* [47], *I*. *cathayensis* [82] and *I*. *loczyi* [87]. Flavanone 5,3,3′‐trihydroxy‐7,4′‐dimethoxyflavanone **66** was first isolated from *I*. *potaninii* rhizomes [56] and *I*.* × germanica* [64]. Additionally, *I*.* × germanica* rhizomes yielded 6‐hydroxy‐5,7,3′,4′,5′‐pentamethoxyflavanone **67** under oxygen deprivation conditions [80], as well as a novel flavanone, 5,7,2′‐trihydroxy‐6‐methoxyflavanone **68** [88].

Of particular interest are flavanones that have an oxidised substituent at the C‐2′ position of the B‐ring. Such compounds are rare in nature and are usually only found in a few plant families, such as Asteraceae, Iridaceae, Fabaceae and Moraceae. Flavanones in *Iris* species are characterised by a higher degree of structural substitution; however, their chemotaxonomic significance and pharmacological profiles remain poorly explored. The rare occurrence of oxygen‐containing substituents on the B‐ring requires further study, especially among taxa belonging to section *Tenuifoliae*.

### 5.4 Peltogynoids

**FIGURE 7:**
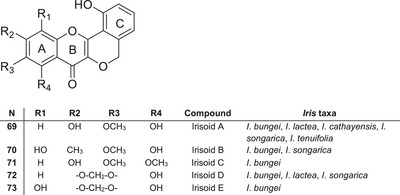
Peltogynoids identified in species of the genus *Iris*.

Irisoids A–E **69–73** were isolated from the underground parts of *I*. *bungei* [48]. These compounds belong to the rare class of flavonoids known as peltoginoids, which contain an additional oxygen‐containing ring (Figure 7). In addition to *I*. *bungei*, irisoid A **69** has also been isolated from five *Iris* species belonging to the section *Tenuifoliae*, such as *I*. *tenuifolia*, *I*. *songarica*, *I*. *cathayensis*, *I*. *loczyi* and *I*. *lactea* [39], suggesting its potential chemotaxonomic significance as a marker compound for this group.

Analysis of the available data indicates that peltogynoids are restricted to a limited number of species within the section *Tenuifoliae*, highlighting their potential value as chemotaxonomic markers. However, due to the limited number of reports, further investigation is required to clarify their distribution, biosynthesis pathway and biological significance.

### 5.5 Coumaronochromones and Rotenoids

**FIGURE 8:**
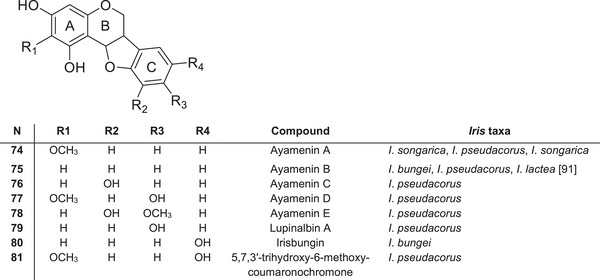
Coumaronochromones (pterocarpans) identified in species of the *Iris* genus.

*Iris* species contain isoflavonoids and flavonoids, some of which have additional five‐ or six‐membered, oxygen‐containing heterocycles in their structure. Coumaronochromones, such as ayamenins A–E **74–78**, have been isolated from the leaves of *I*. *pseudacorus* as stress‐induced metabolites [84, 89] (Figure 8). Ayamenin B **75** and a new coumaronochromone, irisbungin (5,7,5′‐trihydroxy‐coumaronochromone) **80**, were isolated from the roots of *I*. *bungei* [90]. Ayamenin A **74** has also been found in *I*. *songarica* [77] and *I*. *lactea* [91]. Pyranocoumaronochromones, such as lupinalbin **79** A and 5,7,3′‐trihydroxy‐6‐methoxycoumaronochromone **81**, were identified in *I*. *pseudacorus* leaves treated with cupric chloride, which induces the production of fungitoxic stress metabolites [89]. These oxygen‐bridged flavonoid derivatives further expand the structural diversity to specialised metabolites in the *Iris* genus and may play roles in stress tolerance and species‐specific biological activity.

**FIGURE 9:**
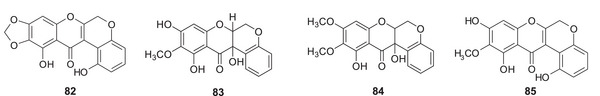
Rotenoids identified in *Iris* species: 1,11‐dihydroxy‐9,10‐methylenedioxy‐12‐dehydrorotenoid **82** (found in *Iris spuria*) and crocetenone **83** (found in *Iris crocea*).

All these species belong to the subgenus *Limniris*. The global literature contains no evidence for the occurrence of coumaronochromones in plants belonging to other subgenera of the *Iris* genus. This suggests that coumaronochromones may be important chemotaxonomic markers for subgenus *Limniris*. In the rhizomes of *I*. *crocea* [85] and *I*. *spuria* [92], which belong to the subgenus *Limniris* ser. *Spuriae*, new 12α‐hydroxyrotenoids were identified: 1,11‐dihydroxy‐9,10‐methylenedioxy‐ **82**, irispurinol (9,11,12‐trihydroxy‐10‐methoxy‐) **83** and 9‐methoxyirispurinol **84** (Figure 9). The new rotenoid crocetenone **85** (9,11‐dihydro‐10‐methoxy‐) was also isolated from the methanolic extract of *I*. *crocea* rhizomes [86]. These compounds, which are absent in other *Iris* taxa, may therefore represent a distinctive chemotaxonomic feature of ser. *Spuriae* within subg. *Limniris*.

These rare flavonoid derivatives are mostly restricted to the subgenus *Limniris*, especially in species exposed to abiotic stress. Their presence suggests potential adaptive significance; however, their ecological functions and therapeutic potential remain largely speculative and should be addressed in future targeted bioactivity studies.

### 5.6 Anthocyanins

**FIGURE 10:**
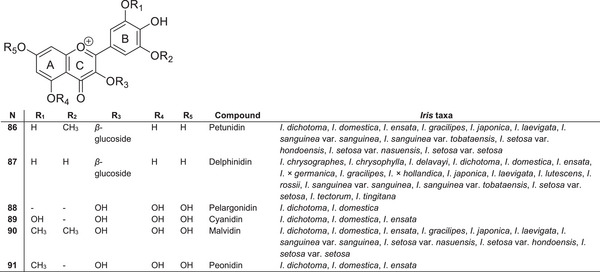
Structural formulas of the most common anthocyanins identified in species of the *Iris* genus.

The variety of perianth colour in species of the *Iris* genus is largely determined by anthocyanins. These plants are characterised by the presence of both leucoanthocyanidins (leucocyanidin and leucodelphinidin) and a diverse array of anthocyanins (Figure 10). Common anthocyanin structures in *Iris* species include delphinidin **87**, petunidin **86** and malvidin **90** derivatives, such as malvidin 3‐*O*‐(*p*‐coumaroylrutinoside)‐5‐glucosides, delphinidin 3‐*O*‐rutinoside‐5‐glucosides and peonidin 3‐*O*‐(*p*‐coumaroylrutinoside) (Figure 9). The most extensively studied anthocyanins have been isolated from *I*. *ensata* [93, 94]. The aerial parts (flowers and leaves) of *I*. *ensata*, *Iris laevigata*, *Iris setosa*, *Iris japonica* and others contain malvidin derivatives, petunidin derivatives, peonidin, cyanidin and their glycosides [39]. Anthocyanins derived from pelargonidin have been identified in the flowers of *I*. *dichotoma* and *I*. *domestica* [95]. These compounds have not been reported in other *Iris* species.

### 5.7 Xanthones

**FIGURE 11:**
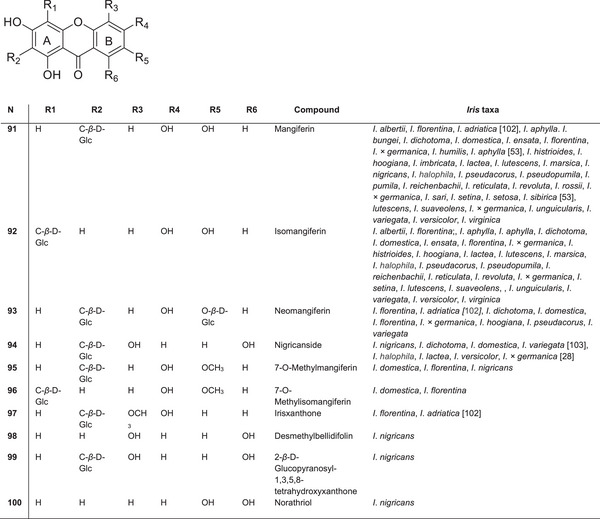
The most common xanthones identified in the *Iris* genus.

The most common and well‐known C‐glycosylxanthone found in *Iris* species is mangiferin **92** (Figure 11). Mangiferin was first identified in the leaves of *Mangifera indica* L. (Anacardiaceae) [96] and has since been detected in several ferns and dicotyledonous plants across at least 28 genera in 19 families. Among monocotyledonous plants, mangiferin was initially discovered in the flowers of *I*.* × germanica* in 1963 and later in other *Iris* species [97].

Mangiferin is widely distributed throughout the genus *Iris*. By the late 1980s, it had been identified in all 15 species of *Iris* from subgenus *Iris* [98]. It was also found in *Iris humilis* (sect. *Psammiris*) and *Iris hoogiana* (sect. *Regelia*). Within subg. *Limniris*, sect. *Limniris*, mangiferin is present in ser. *Tripetalae*, ser. *Laevigatae*, ser. *Ensatae* and ser. *Unguiculares*. In the subg. *Hermodactyloides*, mangiferin has been found in two species: *I*. *histriodes* and *Iris reticulata* [33, 39].

In addition to mangiferin and its isomer isomangiferin **93**, their derivatives, such as irixanthone, 5‐methyl ether, *O*‐methyl ether and O‐glucoside, are also commonly found. These compounds serve as important taxonomic markers for diploid species within the bearded *Iris* group. In 1973, Takemoto's team first isolated irisxanthone from *I*. *florentina* [44]. Compared to mangiferin, irixanthone has a methoxy group at Position 5 and lacks a hydroxyl group at Position 7. Irisxanthone has also been identified in the leaves of *I*. *florentina*, *I*. *adriatica* and *I*.* × germanica* [39].

According to Iwashina and Mizuno [39], mangiferin has been identified in 47 species and subspecies of the *Iris* genus, whereas isomangiferin was found in 41. Among unusual C‐glucosyl xanthones, 6‐dehydrojacareubins were isolated as stress metabolites from *I*.* × germanica* leaves [80], but they have not been reported in other *Iris* species.

New xanthones, including the 1,3,5,8‐tetrahydroxy derivatives bellidifolin and desmethylbellidifolin, as well as nigricanside, were isolated from the rhizomes of *I*. *nigricans* [99]. Another compound, 2‐β‐d‐glucopyranosyl‐1,3,5,8‐tetrahydroxyxanthone, was also found. Mangiferin 7‐*O*‐β‐glucoside (neomangiferin) and nigricanside were also detected in *I*. *dichotoma* [58] and *I*. *domestica* [64], whereas 6′‐*O*‐acetyl mangiferin was found in the rhizomes of *I*. *rossii* [74]. Chinese researchers have indicated the chemotaxonomic significance of mangiferin for the Iridaceae family at multiple taxonomic levels, including tribe, subgenus, section and series [100]. For instance, the *Irideae* and *Tigridae* tribes can be distinguished from other Iridaceae tribes on their mangiferin content [31, 101]. Overall, mangiferin and its derivatives are widely distributed across the *Iris* genus and demonstrate significant potential both as chemotaxonomic markers and as bioactive compounds with anti‐inflammatory, antimicrobial and antiviral properties.

Xanthones, especially mangiferin and its derivatives, are widely distributed across *Iris* species and hold both taxonomic and therapeutic significance. However, the impact of environmental factors on the accumulation of xanthones in *Iris* plants remains poorly understood and requires further study.

### 5.8 Benzophenones and Furans

Benzophenones serve as a key intermediate in xanthone biosynthesis and have been found in several *Iris* species. Iriflophenone is the most common form, occurring in *I*. *adriatica* [102], *I. humilis, I. pumila, I. veriegata* [103], *I*. *florentina* [104, 105], *I*. *halophila*, *I*. *spuria* subsp. *Carthaliniae*, and *I*. *versicolor* [28], *I*. *potaninii* [56], *I*. *scariosa* [106] and *I*. *domestica* [107]. Other derivatives, such as 2,6,4′‐trihydroxy‐4‐methoxybenzophenone, have been identified in *I*.* × germanica* and *I*. *halophila* resinoids [66], as well as in *I*. *adriatica* rhizomes [102]. Additionally, 2,4′,6‐trihydroxy‐4‐methoxybenzophenone‐2‐*O*‐β‐d‐glucoside was isolated from *I*.* × germanica* rhizomes [64].

From *I*. *domestica* seeds, four new enamides, belamcandines A–D, were isolated and identified as 4a,8‐dihydroxy‐2,7‐dimethoxy‐1,4‐dioxo‐1,4,4a,9b‐tetrahydrodibenzofurans with two alkyl side chains at Positions 9 and 9b [108]. Subsequently, the sucrose derivatives belamcanoside A and B and the pyrrole derivative 4‐(2‐formyl‐5‐hydroxymethylpyrrol‐1‐yl) butyric acid were also isolated from the *I*. *domestica* seeds and *I*. *spuria* rhizomes [60, 109], respectively. Overall, benzophenones and furan derivatives remain poorly studied. Given their restricted occurrence, they may serve as promising chemotaxonomic or pharmacological markers upon further investigation.

### 5.9 Quinones and Benzoquinones

One of the earliest reports of quinone isolation from *Iris* rhizomes was by Wong et al. [110]. They found 2‐hydroxy‐3‐octadecyl‐5‐methoxy‐1,4‐benzoquinone (irisoquin) and 3‐octadecyl‐5‐methoxy‐1,4‐benzoquinone (deoxyirisoquin) in *Iris missouriensis*. Irisoquin was later also found in the rhizomes of *Iris kemaonensis* [111] and in the seed oil of *I*. *missouriensis* [112]. Seven homologues, including irisquinone (pallason A), 2‐methoxy‐6‐[*Z*]‐pentadec‐8‐enyl‐1,4‐benzoquinone and 5‐hydroxy‐2‐methoxy‐6‐(pentadec‐7‐enyl)‐1,4‐benzoquinone, were found in the seed oils of *I*. *pseudacorus*, *I*. *sibirica* and *I*. *missouriensis* [112]. Pallason A, B (dihydroirisquinone) and C were isolated from *I*. *lactea* [113].

Several alkylated p‐benzoquinones have been reported in other species. For example, irisoquin A–F were identified in *I*. *kemaonensis* rhizomes [111], 3‐[(*z*)‐12′‐heptadecenyl]‐2‐hydroxy‐5‐methoxy‐1,4‐benzoquinone in the same species [114] and nepaloquinone A in *I*.* × germanica* rhizomes [115]. From *I*. *bungei* seeds and roots, novel derivatives such as belamcandaquinone N, 3‐hydroxyirisquinone, bungeiquinone and dihydrobungeiquinone were also isolated [116, 117]. Other benzoquinones, including irisquin B, 3‐hydroxyirisquinone and 2‐acetoxy‐3,6‐dimethoxy‐1,4‐benzoquinone, were found in *I*. *crocea*, *Iris suaveolens* and *I*.* × germanica*, respectively [85, 118, 119].

Additionally, Tie et al. [120] described a new oligostilbene, vitisin A‐13‐*O‐*β‐d‐glucoside, alongside known oligostilbenes vitisin D, hopeaphenol and vitisin A, all from *I*. *lactea* seeds. The abundance of these quinones and oligostilbenes in rhizomes and seeds highlights their structural diversity, species specificity and potential roles in plant defence and stress adaptation, as well as their promising pharmacological applications.

### 5.10 Chromanes and Chromones

Chromane, also known as benzo‐γ‐dihydropyran, is a heterocyclic compound that serves as a structural scaffold for more complex molecules, including vitamin E (tocopherols and tocotrienols). For the first time, Dagvadorj's working group in Mongolia [121] isolated five new compounds from the underground parts of *I*. *tenuifolia*, including an unusual macrolide, moniristenulide. Additionally, the chromone derivative 5,7‐dihydroxy‐6‐methoxychromone was identified in the rhizomes of *I*. *unguicularis* [122], further expanding the diversity of heterocyclic metabolites in the genus.

Chromanes and chromones are relatively rare in the *Iris* genus and have been reported in only a few taxa, often as minor components. Their limited distribution suggests potential chemosystematic significance, but comprehensive screening across more species is needed to clarify their taxonomic and biological relevance.

### 5.11 Benzenecarboxylic Acids, Ketones, Phenylpropanoids

Hydroxybenzoic acids are common precursors in the biosynthesis of flavonoids and isoflavonoids, particularly in *Iris* species. Among these, gallic acid, *p*‐hydroxybenzoic acid, protocatechuic acid, vanillic acid and syringic acid have been frequently identified. Hydroxycinnamic acids are also widely distributed and include ferulic acid, caffeic acid, *p*‐coumaric acid, *trans*‐cinnamic acid, chlorogenic acid, neochlorogenic acid and sinapic acid [28, 29, 30, 31, 32, 33, 34, 35, 36, 37, 38, 39, 40, 41, 42, 43, 44, 45, 46, 47, 48, 49, 50, 51, 52, 53, 54, 55, 56, 57, 58, 59, 60, 61, 62, 63, 64, 65, 66, 67, 68, 69, 70, 71, 72, 73, 74, 75, 76, 77, 78, 79, 80, 81, 82, 83, 84, 85, 86, 87, 88, 89, 90, 91, 92, 93, 94, 95, 96, 97, 98, 99, 100, 101, 102, 103, 104, 105, 106, 107, 108, 109, 110, 111, 112, 113, 114, 115, 116, 117, 118, 119, 120, 121, 122, 123, 124, 125, 126]. These phenolic acids contribute to the structural diversity of downstream secondary metabolites and may play roles in plant defence, stress response and pigmentation (Tables 2 and 3).

**TABLE 2:** The derivatives of phenolcarboxylic acids identified in *Iris* taxa.

Compound	*Iris* taxa
Vanillic acid	*I*. *bungei* [90]; *I*. *dichotoma*, *I*. *humilis*, *I*. *lactea*, *I*. *tenuifolia* [125]; *I*. *halophila*, *I*. *versicolor*, *I*. *lactea*, *I*. *spuria* subsp. *Carthaliniae*, *I*.* × germanica* [28]; *I*. *spuria [* 60]
Vanillic acid 4‐*O*‐β‐d‐glucopyranoside	*I*. *spuria* [60]
Syringic acid	*I*. *bungei* [90]; *I*. *humilis*, *I*. *lactea* [125]; *I*. *schachtii* [126]
Glucosyringic acid	*I*. *spuria* [60]
Gallic acid	*I*. *domestica* [59]; *I*. *pseudacorus*, *I*. *sibirica*, *I*.* × germanica*, *I*. *variegata* [57]; *I*. *aphylla* [53]; *I*. *lactea* [125]; *I*. *schachtii* [126]
Protocatechuic acid	*I*.* × germanica* [127]; *I*. *dichotoma*, *I*. *humilis*, *I*. *bungei*, *I*. *lactea*, *I*. *tenuifolia* [125]; *I*. *schachtii* [126]; *I*. *halophila*, *I*. *versicolor*, *I*. *lactea*, *I*. *spuria* subsp. *carthaliniae*, *I*.* × germanica* [28]
3‐Hydroxybenzoic acid	*I*. *bungei*, *I*. *tenuifolia* [125]
4‐Hydroxy benzoic acid	*I*.* × germanica* [127]; *I*. *dichotoma*, *I*. *humilis*, *I*. *bungei*, *I*. *lactea*, *I*. *tenuifolia* [125]; *I*. *schachtii* [126]; *I*. *halophila*, *I*. *versicolor* [28]; *I*. *domestica* [59]
Benzoic acid	*I*. *schachtii* [126]
Caffeic acid	*I*. *aphylla* [124]; *I*. *pseudacorus* [123]; *I*. *sibirica* [52]; *I*.* × germanica*, *I*. *variegata* [53]; *I*. *halophila* [127]
Chlorogenic acid	*I*. *aphylla* [124]; *I*. *pseudacorus*, *I*. *sibirica* [52]; *I*. *germanica* [127]
Neochlorogenic acid	*I*. *aphylla* [124]; *I*. *pseudacorus*, *I*. *sibirica*, *I*.* × germanica* [52]
Ferulic acid	*I*. *aphylla* [124]; *I*. *pseudacorus*, *I*. *sibirica* [52]; *I*.* × germanica* [127]; *I*. *dichotoma*, *I*. *humilis*, *I*. *lactea* [125] *I*. *halophila*, *I*. *versicolor*, *I*. *lactea*, *I*. *spuria* subsp. *carthaliniae*, *I*.* × germanica* [28]; *I*. *nigricans* [45]
*p*‐Coumaric acid	*I*. *pseudacorus*, *I*. *sibirica*, *I*.* × germanica* [52]; *I*. *humilis*, *I*. *lactea* [125]; *I*. *schachtii* [126]
*trans*‐Cinnamic acid	*I*. *pseudacorus*, *I*. *sibirica*, *I*.* × germanica*, *I*. *halophila* [52]; *I*. *dichotoma*, *I*. *humilis*, *I*. *bungei*, *I*. *lactea*, *I*. *tenuifolia* [125]; *I*. *schachtii* [126]; *I*. *halophila*, *I*. *versicolor*, *I*. *lactea*, *I*. *spuria* subsp. *carthaliniae*, *I*.* × germanica* [28]
Sinapic acid	*I*. *schachtii* [126]
Shikimic acid	*I*. *domestica* [59]

**TABLE 3:** Acetophenones derivatives identified in *Iris* taxa.

3‐Hydroxy‐5‐methoxyacetophenone	*I*.* × germanica* [88]
Apocynin (4‐hydroxy‐3‐methoxyacetophenone)	*I*.* × germanica* [119]; *I*. *pallida* [132]; *I*. *suaveolens* [129]; *I*. *nigricans* [45]; *I*. *tectorum* [133, 134]; *I*.* × germanica* [64]; *I*. *tectorum* [135]; *I*. *domestica* [136]; *I*. *pallida* [132]; *I*. *tingitana* ([131]; *I*. *crocea* [86]
Androsin (4‐*O*‐β‐d‐glucopyranosyl‐acetovanillone)	*I*. *postii* [137]; *I*. *tectorum* [133]; *I*.* × germanica* [64]; *I*. *postii* [137]; *I*. *tectorum* [135]
Tectorusid	*I*. *tectorum* [134]; *I*. *domestica* [138]
Diapocynin	*I*. *tectorum* [134]
Apocynin‐4‐*O*‐β‐d‐(6′‐*O*‐syringyl) glucopyranoside	*I*. *tectorum* ([135]
Scrophenoside C‐7‐ethylether	*I*. *tectorum* [135]
Apocynin‐4‐*O*‐β‐d‐xylopyranoside	*I*. *tectorum* [139]

The phenylpropanoid glycoside e‐coniferin has been identified in *I*. *spuria* [128]. In additional, an unusual phenylpropanoid containing an epoxy group, *cis*‐epoxyconiferyl alcohol, was isolated from the fresh rhizomes of *I*. *suaveolens*, alongside coniferaldehyde and p‐hydroxyacetophenol [129].

Acetovanillone (apocynin), its β‐d‐glucoside isorhizone and tectoroside were identified in the roots of *I*. *tectorum* [90]. Apocynin has also been found in *I*.* × germanica*, *I*. *suaveolens*, *I*. *nigricans*, *I*. *susiana* and *I*. *tingitana* [45, 46, 129, 130, 131]. Overall, benzenecarboxylic acids and phenylpropanoids are widespread in *Iris* species, though their ecological roles remain largely unexplored.

### 5.12 Phytosterols

Steroids are also present in the rhizomes of *Iris* species, though they are less abundant than phenolic compounds. Among the identified steroidal compounds, β‐sitosterol has been reported in *I*. *tenuifolia* [47], *I*.* × germanica* [64], *I*. *lactea* [91], *I*. *susiana* [140], *I*. *suaveolens* [118] and *I*. *domestica* [141]. Other steroids include daucosterol [64], stigmasterol and stigmasterol‐3‐*O*‐β‐d‐glucopyranoside [88] in *I*.* × germanica*, as well as 7‐β‐hydroxystigmast‐4‐en‐3‐one in *I*. *suaveolens* [118]. Isoarborinol and stigmasterol were isolated from the underground parts of *I*. *confusa* [6]. Daucosterol was isolated from *I*. *domestica* [141] and *I*. *lactea* [91]. Tristearoyl‐sn‐glycerol, 1‐margaroyl‐2‐lauroyl‐3‐palmitoyl‐sn‐glycerol and 1,2‐distearoyl‐sn‐glycerol were isolated from *I*. *marsica* [67]. Stigmasterol was isolated from *I*. *susiana* [140]. A complete overview of the phytosterols isolated or identified in *Iris* species is provided in Table 4.

**TABLE 4:** Stilbenes identified in *Iris* taxa.

Compound	*Iris* taxa
Stigmasterol	*I*.* × germanica* [88]; *I*. *susiana* [46]; *I*. *cathayensis* [82]; *I*. *songarica* [142]
Stigmasterol‐3‐*O*‐β‐d‐glucopyranoside	*I*.* × germanica* [88]
β‐Sitosterol	*I*. *susiana* [46]; *I*. *tenuifolia* [47]; *I*. *suaveolens* [129]; *I*. *lactea* [91]; *I*. *domestica* [141]; *I*. *adriatica* [104]; *I*. *leptophylla* [55]; *I*. *songarica* [142]; *I*. *tectorum* [133]; *I*.* × germanica* [64]; *I*. *florentina* [44]; *I*. *nigricans* [45]
Dausterol	*I*. *domestica* [59]; *I*. *lactea* [91]; *I*. *halophila* var. *sogdiana* [106]; *I*. *cathayensis* [82]; *I*. *leptophylla* [55]; *I*. *songarica* [142]; *I*. *tectorum* [133]; *I*.* × germanica* [64]
Resveratrol	*I*. *hookeriana* [143]; *I*. *dichotoma* [58]; *I*. *domestica* [144]; *I*. *halophila* [145]; *I*. *clarkei* [146]
Resveratroloside (*trans*‐resveratrol 4′‐*O*‐β‐d‐glucuronide)	*I*. *hookeriana* [143]
*trans*‐Resveratrol 3‐*O*‐β‐glucoside	*I*. *tingitana* [60]
Resveratrol 3,4′‐*O*‐di‐β‐d‐glucopyranoside	*I*. *postii* [137]
Piceid	*I*. *hookeriana* [143]; *I*.* × germanica* [85]
Ampelopsin B	*I*. *clarkei* [146]
(+)‐α‐Viniferin	*I*. *clarkei* [146]
(−)‐*trans‐*ε‐Viniferin	*I*. *halophila* [145]; *I*. *lactea* [147]; *I*. *postii* [137]
*trans*‐ε‐Viniferin‐13b‐β‐d‐glucopyranose	*I*. *lactea* [147]
*cis*‐ε‐Viniferin‐11a,13b‐di‐*O*‐β‐d‐glucopyranoside	*I*. *lactea* [147]
γ‐2‐Viniferin	*I*. *halophila* [145]
Halophilol A	*I*. *halophila* [145]
Halophilol B	*I*. *halophila* [145]
Vatalbinoside C	*I*. *lactea* [147]
*cis*‐Vitisin B‐13b‐*O*‐β‐d‐glucopyranoside	*I*. *lactea* [147]
*cis*‐Vitisin B	*I*. *lactea* [147]
*cis*‐Vitisin C	*I*. *lactea* [147]
Vitisin A	*I*. *lactea* [147]
Vitisin B	*I*. *lactea* [147]
(−)‐Hopeaphenol	*I*. *domestica* [109]
7β‐Hydroxystigmast‐4‐en‐3‐one	*I*. *suaveolens* [129]
Tingitanol A	*I*. *tingitana* [60]
Tingitanol B	*I*. *tingitana* [60]

Some species in the *Iris* genus produce both monomeric and oligomeric stilbenes. Resveratrol has been identified in *I*. *dichotoma* [58], *I*. *domestica* [144], *Iris hookeriana* [143], *I*. *halophila* [145] and *Iris clarkei* [146]. The resveratrol glucosides piceid (3‐*O*‐β‐d‐glucoside of resveratrol) and resveratroloside were isolated from the underground organs of *I*. *hookeriana* [143], with piceid also detected in *I*.* × germanica* [85]. Two stilbenes containing a γ‐lactam unit [(+) and (−)] were isolated from the rhizomes of *I*. *domestica* [65]. Additionally, resveratrol‐3‐glucoside and two new stilbene glycosides, tingitanols A and B, were isolated from *I*. *tingitana* (subg. *Xiphium*) [60].

Monomeric stilbene halophylol A and the tetrameric stilbene halophylol B were found in the seeds of *I*. *halophila* (subg. *Limniris*, sect. *Limniris*), along with *ε*‐viniferin and γ‐2‐viniferin [145]. Two resveratrol‐type oligostilbenes, ampelopsin B and α‐viniferin, were isolated from the seeds of *I*. *clarkei* [146]. Ten stilbene oligomers, including vatalbinoside C, *cis*‐vitisin B‐13b‐*O*‐β‐d‐glucopyranoside, *trans*‐ε‐viniferin‐13b‐β‐d‐glucopyranose, *cis‐ε*‐viniferin‐11a,13b‐di‐*O*‐β‐d‐glucopyranoside, *trans‐*ε‐viniferin, *cis*‐vitisin B and C and vitisin A and B, were also found in *I*. *lactea* seeds [147]. Furthermore, *trans‐ε*‐viniferin and resveratrol 3,4′‐*O*‐di‐β‐d‐glucopyranoside were isolated from *Iris postii* [137], and the stilbenoid (−)‐hopeaphenol, a hydroxylated derivative of stilbene, was isolated from *I*. *domestica* seeds [109]. These are the only examples of the isolation of oligomeric stilbenes from the Iridaceae family.

Phytosterols have been sparsely identified in *Iris* species, mainly in rhizomes, and have not been comprehensively assessed. Comparative profiling across species and developmental stages may reveal taxon‐specific sterol patterns and support their evaluation as bioactive or chemotaxonomic markers.

### 5.13 Alkaloids

Data on the presence of alkaloids in *Iris* species are extremely limited. The notable exception is the study by Xie et al. [148], who investigated the ethanolic extract of *I*.* × germanica* rhizomes and identified several alkaloid compound structures. These included 1,2,3,4‐tetrahydro‐c‐carboline‐3‐carboxylic acid, *S*‐(−)‐methyl‐1,2,3,4‐tetrahydro‐9*H*‐pyrido[3,4‐b]indole‐3‐carboxylate, (1*R*,3*R*)‐methyl‐1‐methyl‐2,3,4,9‐tetrahydro‐1*H*‐pyrido[3,4‐b]indole‐3‐carboxylate, (1*S*,3*R*)‐methyl‐1‐methyl‐2,3,4,9‐tetrahydro‐1*H*‐pyrido‐[3,4‐b]indole‐3‐carboxylate, 4‐(9*H*‐c‐carbolin‐1‐yl)‐4‐oxobut‐2‐enoic acid methyl ester, 2‐(furan‐2‐yl)‐5‐(2,3,4‐trihydroxybutyl)‐1,4‐diazine, 3‐c‐d‐ribofuranosyluracil,6‐hydroxymethyl‐3‐pyridinol and 2‐amino‐1H‐imidazo[4,5‐b]pyrazine. The findings of the study represent the first detailed characterisation of alkaloids in *Iris* genus, particularly in *I*.* × germanica*. Given the scarcity of such data, further phytochemical investigations across additional *Iris* species are warranted to explore the potential chemotaxonomic relevance and potential pharmacological properties of their alkaloid profiles.

### 5.14 Terpenoids

Iridals constitute a distinct family of triterpenoids that are the primary lipid components of *Iris* rhizomes. These compounds are typically monocyclic, featuring a modified ring A with various degrees of unsaturation and/or hydroxylation, or they may occur as bicyclic C_31_‐triterpenoids. The latter class arises through cyclisation of the homofarnesyl side chain, initiated by the transfer of a methyl group from S‐adenosylmethionine to the terminal double bond. The resulting cycloiridals act as biosynthetic precursors of dihydroirones and irones [149, 150]. Notably, no single terpenoid has been reported to occur in more than one *Iris* taxon. Therefore, the information in Table 5 is arranged according to species, from *Iris* taxa to the corresponding identified compounds.

**TABLE 5:** Iridal‐type triterpenoids identified in species of the genus *Iris*.

*Iris* taxa	Triterpenoid	Refs.
*I*. *delavayi*	6*S*,10*R*,11*R*)‐18,19‐Epoxy‐10‐deoxy‐17‐hydroxyiridal, (2(7)E,6*S*,10*R*,11*R*)‐10‐Deoxy‐17‐hydroxyirida, (2(7)*E*,6*S*, 10*R*, 11*R*)‐18,19‐Epoxy‐10‐deoxy‐17‐hydroxyiridal	[151]
*I*. *confusa*	Spirioiridoconfal A‐C, isobelamcandal, 17‐hydroxyl‐27‐ene‐iridal	[152]
*I*. *forrestii*	Forrestin A, forrestin B	[153]
*I*. *foetidissima*	6*R*,10*S*,11*S*,14*S*,26*R*)‐26‐Hydroxy‐15‐methylidenespiroirid‐16‐en	[150]
*I*. *tectorum*	Iritectol G, Iritectol H, 19‐epiiritectol H	[154]
*I*. *tectorum*	Iritectorol A, iritectorol B, iridobelamal A, isoiridogermanal	[155]
*I*. *tectorum*	Iridotectoral A, iridotectoral B, 28‐deacetylbelamcandal, (6*R*,10*S*,11*R*)‐26‐x‐hydroxy‐(13*R*)‐oxaspiroirid‐16‐enal	[156]
*I*. *tectorum*	Iridotectoral C, Iridotectoral D	[157]
*I*. *tectorum*	Polycycloiridals A–D	[158]
*I*. *tectorum*	Spirioiridotectals A−F	[158]
*I*. *domestica*	Iridobelamal B	[157]
*I*. *domestica*	Iridobelamal A, 28‐deacetylbelamcandal, (6*R*,10*S*,11*R*)‐26‐x‐hydroxy‐(13*R*)‐oxaspiroirid‐16‐enal, 6*R*,10*S*,11*S*,14*S*,26*R*)‐26‐hydroxy‐15‐methylidene‐spiroirid‐16‐enal, 16‐*O*‐acetyl‐iso‐iridogermanal, isoiridogermanal	[156]
*I*. *domestica*	Polycycloiridals K–T	[159]
*I*. *domestica*	Belamchinenin A	[160]
*I*. *domestica*	Dibelamcandal A	[161]
*I*. *domestica*	16‐Acetyliridal A, 16‐acetyliridal B	[162]
*I*. *tenuifolia*	Arborinone	[47]
*I*. *variegata*	21‐Hydroxyiridal, 21‐hydroxy‐10‐deoxyiridal, 16‐hydroxyiridal, 22‐methyl‐γ‐cycloiridal, 23‐hydroxyiridal	[149]
*I*.* × germanica*	Isoiridogermanal, 16‐*O*‐acetylisoiridogermanal, α‐iridogermanal, γ‐iridogermanal, α‐dehydroiridogermanal, iridial, iriflorental, iripallidal, irisgermanicals A–C	[163, 164]
*I*.* × germanica*	Irigermanal, iridogermanal	[165]
*I*.* × germanica*	Iristectoron K	[51]
*I*. *crocea*	Iridalglycoside 5b, iridalglycoside 7	[85]
*I*. *spuria*	Iridalglycoside 5b/6c, iridalglycoside 7/8	[85]
*I*. *pseudacorus*	Irispseudoacorins A–D, 16‐formyl‐isoiridogermananl, 3‐formyl‐isoiridogermananl, iridojaponals A‐B, isoiridogermananl; iridobelamal A; 17(*R*)‐hydroxyliridal	[166]
*I*. *versicolor*	22,23‐Dihydro‐22‐methyleneiridal, 26‐hydroxyiridal, 6‐methylhept‐5‐en‐2‐one, (4*RS*)‐4‐hydroxy‐6,1O‐dimethylundeca‐5,9‐dien‐2‐one, 17*ξ*,26‐dihydroxyiridal	[167]
*I*. *sibirica*	Iridal, spirocyclic hemiacetal, 16*ξ*‐hydroxyiridal, 26‐hydroxyiridal, 17*ξ*,26‐dihydroxyiridal, 17*ξ*‐hydroxyiridal, 10‐deoxy‐l7*ξ*‐hydroxyirid	[167]
*I*. *sibirica*	(6*R*,10*S*, 11*S*)‐17,29‐Didehydroiridal	[168]

Pentacyclic triterpenes, such as ursolic acid, betulin, betulonic acid and betulone, have been identified in the rhizomes of *I*. *domestica* [141]. Arborinone, a 3‐oxygenated pentacyclic triterpene, was isolated from *I*. *tenuifolia* [47]. Although most reviews of Iris phytochemistry emphasise isoflavonoids and xanthones, triterpenoids, particularly those occurring in the rhizomes, also present a promising but understudied class of compounds.

Terpenoids, especially triterpenoids and iridals, constitute major secondary metabolites in *Iris* rhizomes. Although some of them have demonstrated cytotoxic, anti‐inflammatory and antimicrobial activities, their structural diversity and biological functions remain largely unexplored. Comprehensive metabolomic and genomic investigations are therefore required to clarify their biosynthetic origins and assess their potential therapeutic applications.

### 5.15 Essential Oil

The industrial production of *Iris* essential oil for perfumery, at a concentration of about 0.1%–2.0%, began in 1912 at the Santa Maria Novella factory in France. Initial production volume was 10 kg per year. The cultivation of *Iris* species for industrial purposes, however, dates back to the mid‐19th century in Italy [169]. Today, only a few regions worldwide cultivate *Iris* species for use as a plant raw material in the perfume industry: *I*. *pallida*, *I*.* × germanica* and *I*. *florentina* in Tuscany and Florence (Italy); *I*. *pallida* in Landes (France); and *I*.* × germanica* in Marrakech, in the foothills of the Atlas Mountains (Morocco).

Orris oil, valued for its characteristic violet fragrance, is obtained from the rhizomes of *I*. *florentina*, *I*.* × germanica* and *I*. *pallida*. The crude extract appears as a semi‐solid yellow mass known as *Iris concrete*. Essential oil have also been identified in the rhizomes and leaves of several *Iris* species, including *I*. *persica* [170], *I*. *variegata* [149], *I*. *bulleyana* [171], *I*. *aphylla* [172], *I*. *medwedewii*, *I*. *spuria* subsp. *carthaliniae* [173], *I*. *versicolor*, *I*. *graminea*, *I*. *halophila* [174], *I*. *haussknechtii*, *I*. *susiana* [175], *I*. *nigricans* [176], *I*. *taochia* [177], *I*. *planifolia* [178], *I*. *pseudopallida*, *I*. *illyrica* and *I*. *adriatica* [179].

**FIGURE 12:**
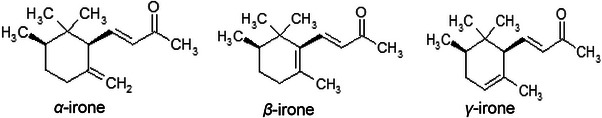
The main components of *Iris* essential oil.

The essential oils of *Iris* species contain a complex mixture of volatile organic compounds, including monoterpenes, sesquiterpenes, diterpenes, triterpenes, fatty acids, aliphatic hydrocarbons and aldehydes. The monoterpene ketone α‐irone and the triterpenoid squalene have been identified in all analysed samples, making them potential chemotaxonomic and chemosystematic markers for the genus *Iris* [180]. *α*‐Irone, which accounts for 10%–20% of the essential oil and provides its characteristic violet scent, occurs in three structural and stereoisomeric forms: α‐, β‐ and γ‐irone (Figure 12).

Among the components of *Iris* essential oils, various norterpenoids, such as β‐ionone‐5,6‐epoxide, β‐ionone, *trans*‐2,6‐γ‐irone, β‐isometilionone and β‐damascenone, have been identified. Other constituents include neophytadiene, eugenol, α‐terpineol, germacrene D, terpinen‐4‐ol, hexahydrofarnesylacetone, farnesylacetone, phenylacetaldehyde, geranylacetone and 2‐methoxy‐4‐vinylphenol. Essential oils from *Iris* species are also characterised by a high proportion of myristic acid (45%–85%), as well as other fatty acids (caprylic, capric, lauric, palmitic etc.) and their esters.

Essential oils obtained from *Iris* species, especially from the rhizomes, contain unique aromatic compounds such as irones, which are of high value in perfumery and traditional medicine. However, the chemical composition of these oils varies considerably among species and is influenced by environmental conditions, developmental stage and extraction methods. Despite their long‐standing commercial significance, phytochemical profiling of *Iris* essential oils remains incomplete for many taxa. Comprehensive metabolomic studies employing standardised analytical protocols and broader taxonomic coverage are needed to fully elucidate their chemical diversity, biological activities and potential for industrial and pharmacological applications.

### 5.16 Organic and Amino Acids

Various organic acids have been identified in *Iris* species, predominantly in the aerial parts. The chemical composition of the carboxylic acids has been studied in the rhizomes of *I*. *spuria* subsp. *carthaliniae* and *I*. *paradoxa* [181], as well as in the leaves and rhizomes of *I*. *graminea* and *I*. *halophila* [182], and in the leaves of *I*. *aphylla*, *I*.* × germanica*, *I*. *halophila* and *I*. *variegata* [183]. Lactic acid was also found in *I*. *marsica* [67]. These compositions include both short‐ and long‐chain carboxylic acids, such as monocarboxylic, dicarboxylic and tricarboxylic acids, as well as saturated and unsaturated fatty acids.

For the first time, six previously undescribed long‐chain fatty acid esters and isoflavone glycosides were isolated from the rhizomes of *I*. *domestica* [184]. Asghar et al. [185] identified 11 compounds in the petroleum ether extract of *I*.* × germanica* rhizomes, with hexadecanoic and octadecenoic acid methyl esters being the most abundant. Another study [186] reported 23 compounds in *I*.* × germanica* rhizomes, including methyl esters of octadecanoic, palmitic and linolenic acids, as well as lauric, oleic and caprylic acids, among other derivatives. Additional analyses have been conducted on *I*. *sibirica* leaves and rhizomes [187], *I*.* × germanica*, *Iris barnumiae*, *Iris bostrensis* and *Iris aurantica* [188] and *I*. *taochia* [177]. Notably, myristic acid dominated in some species (61%–80%).

Typically, *Iris* leaves exhibit a higher content of unsaturated fatty acids (such as oleic, palmitoleic, linoleic and linolenic acids) than rhizomes, due to their elevated levels of polyunsaturated fatty acids and a lower proportion of saturated (e.g., palmitic, lauric and myristic) and monounsaturated fatty acids. Conversely, rhizomes, as a rule, accumulate more saturated fatty acids while containing less unsaturated ones.

Pyroglutamic acid was found in *I*. *aphylla* rhizomes, whereas alanine, valine, isoleucine, proline, serine, aspartic acid and pyroglutamic acid were found in *I*. *variegata* rhizome extract [189]. Phenylalanine, isoleucine and valine were identified in the rhizomes of *I*. *marsica* [67] and l‐tryptophan was present in the rhizomes of *I*. *postii* [137].

The diversity of carboxylic and amino acids across different species and organs of the *Iris* genus, as well as under various environmental conditions, highlights its value as a source of both primary and secondary metabolites. The organ‐specific distribution pattern, whereby rhizomes accumulate more saturated fatty acids and leaves are enriched in unsaturated forms, underlines the ecological and physiological specialisation of these plants. Continued exploration of *Iris* species phytochemistry reflects the ecological and physiological specialisation of these plants.

Organic and amino acids are essential primary metabolites in *Iris* species, contributing to plant physiology, stress responses and serving as precursors for secondary metabolism. However, their profiles have not been systematically analysed across species or organs, and their chemotaxonomic or ecological significance remains largely unexplored. Targeted metabolomic investigation could elucidate their potential roles in species adaptation, environmental interaction and metabolic specialisation within the genus.

## 6 The Effect of Environmental Factors

The composition of primary and secondary plant metabolites is significantly influenced by environmental factors. Various *Iris* species have been analysed depending on their habitat characteristics, including climate, light intensity, precipitation, soil composition and other ecological parameters. The greatest changes in metabolite profile, particularly in the concentration of isoflavones, hydroxycinnamic acids and xanthones, have been observed among *Iris* species.

Using *I*. *variegata* as an example [190], it was found that the accumulation of these compounds is highest in spring and decreases towards autumn. Plants grown under high light intensity exhibited elevated levels of chlorogenic acid and mangiferin glucoside, whereas caffeic acid, luteolin and naringenin showed the opposite trend. The developmental stage of the growing season strongly influenced metabolite profiles, with almost all secondary metabolites reaching their maximum concentrations in spring and declining progressively during summer and autumn until the end of the vegetative period.

Our previous study [52] demonstrated that the levels of phenolic compounds in *Iris* rhizomes from subgenus *Limniris* and *Iris* were significantly affected by soil nutrient composition. Specifically, the contents of phosphorus and potassium in the soil positively influenced phenolic accumulation, whereas nitrogen levels had no significant effect. Meteorological factors in Ukraine, Lithuania and Latvia had limited impact overall; however, sunshine duration correlated positively, whereas rainfall correlated negatively with phenolic content.

Experimental cultivation of *Iris* species under monocomponent mineral nutrition [191] further confirmed the sensitivity of their secondary metabolism. it is a positive impact on the biosynthesis and accumulation of specific isoflavonoids (e.g., tectoridin, nigricin d‐glucoside, genistin, irisectorigenin B, nigricin, irigenin, irisolidone) and the xanthone mangiferin was observed in rhizomes. Additionally, an increase in chlorogenic acid content was recorded in leaves. These findings indicate that *Iris* phenylpropanoid metabolism responds strongly to mineral nutrition, suggesting that cultivation strategies can be optimised to enhance the production of bioactive compounds.

Although *Iris* species display high ecological plasticity and adaptability to diverse habitats, the environmental factors of their metabolic biosynthesis remain poorly studied. Current evidence suggests that soil composition, climate factors and biotic stressors can significantly influence both the qualitative and quantitative profiles of secondary metabolites. However, comprehensive eco‐chemical correlation studies are still lacking. Future research should focus on evaluating the influence of environmental variables on *Iris* metabolite composition across different geographical regions to better understand species resilience and support the sustainable cultivation of plants with high pharmacological potential.

## 7 Perspectives and Future Directions

The genus *Iris* represents a diverse and important group within the Iridaceae family, characterised by a wide global distribution and a long history of ethnomedicinal use [192]. This review consolidates current data on the primary and secondary metabolites identified in more than 90 taxa, with particular emphasis on isoflavonoids, flavonoids, xanthones and rare compound classes, such as peltogynoids and coumaronochromones. Many of these metabolites exhibit potent pharmacological activities, including antimicrobial, antitumour, antioxidant, antiviral and anti‐inflammatory properties. These findings highlight *Iris* species as promising candidates for natural product‐based drug discovery.

The medicinal potential of *Iris* plants offers valuable opportunities for application in the pharmaceutical, food and cosmetic industries, provided that rigorous quality control of raw materials is maintained throughout cultivation, harvesting and processing [193]. However, not all species within the genus have been studied in detail. Continued efforts to isolate novel bioactive compounds and evaluate their pharmacological properties are warranted, as such studies may yield new therapeutic agents and expand industrial applications.

Despite considerable advances in the phytochemical characterisation of *Iris* species, significant knowledge gaps remain. Of the more than 300 known species, only about one‐third have been chemically characterised, and the majority of studies have focused on a limited number of taxa, primarily *I*.* × germanica*, *I*. *tectorum* and *I*. *dichotoma*. Moreover, the influence of ecological, geographical and environmental factors on metabolite profiles remains insufficiently explored. Future research employing advanced metabolomic, transcriptomic and genomic tools will be essential for elucidating biosynthetic pathways, identifying species‐specific chemical markers and understanding the ecological and evolutionary determinants of secondary metabolism within the genus.

Despite extensive phytochemical research, many *Iris* species remain poorly characterised, especially endemic and geographically restricted taxa. Furthermore, the effects of environmental and ecological factors on metabolite composition are still insufficiently studied. Future investigations should therefore focus on:

- expanding metabolomic profiling to include lesser‐known *Iris* species;
- elucidating the influence of ecological and geographical variation on metabolite accumulation of secondary metabolites;
- integrating omics‐based approaches (e.g., genomics, transcriptomics and metabolomics) to clarify biosynthetic pathways;
- exploring the pharmacological activities of both crude extracts and isolated constituents using relevant biological models.

Overall, *Iris* species emerge as promising plants for advancing the discovery of natural products and chemotaxonomy. Continued interdisciplinary efforts will pave the way for its sustainable use in medicine, cosmetics and biotechnology, while also helping to conserve plant biodiversity.

## Author Contributions

**Olha Mykhailenko**: conceptualisation, data analysis, writing – original draft preparation, writing – review and editing. **Liudas Ivanauskas**: data analysis, writing – review and editing. **Victoriya Georgiyants**: data analysis, writing – original draft preparation, writing – review and editing. **Zigmantas Gudžinskas**: writing – original draft preparation, writing – review and editing. All authors have read and agreed to the published version of the manuscript.

## Conflicts of Interest

The authors declare no conflicts of interest.

## Data Availability

The authors have nothing to report.
